# Editorial: Immune and oxidative pathways in psychiatric disorders

**DOI:** 10.3389/fnins.2022.1073757

**Published:** 2022-10-31

**Authors:** Angela Maria Casaril

**Affiliations:** Laboratories of Neuroimmunology, Department of Symptom Research, The University of Texas MD Anderson Cancer Center, Houston, TX, United States

**Keywords:** inflammation, oxidative stress, resolving D1, mitochondrial, infection, microglia, kynurenine, psychiatry

Psychiatric disorders affect more than 970 million people in the world (Institute of Health Metrics and Evaluation, 2019) and represent a great medical and social challenge. At the social level, people suffering from psychiatric disorders are victims of stigma and discrimination, which significantly impacts their personal and professional lives. At the medical level, the pathological mechanisms are not fully elucidated, which reflects the limited success of treatments available. Notably, psychiatric disorders are associated with complex, heterogeneous, and disease-specific patterns of cognitive dysfunctions, which contribute to severely compromising the patient's quality of life (Millan et al., 2012).

Clinical and preclinical studies have identified that activation of immune and oxidative pathways are intertwined with key pathological factors. Despite the obvious differences in clinical manifestations, mental illnesses such as depression, anxiety, bipolar disorder, post-traumatic stress disorders, addiction, and schizophrenia share common biological alterations, including inflammatory cytokine signaling alteration, microglial activation, increased oxidative stress, disturbance of the blood-brain barrier (BBB), mitochondrial dysfunction, and energy impairment. In this sense, this Research Topic of Frontiers in Neuroscience aimed at bringing together clinical, preclinical, and literature reviews to provide insights into the role of immune and oxidative pathways on the etiology of psychiatric disorders and on the identification of novel molecular targets and pharmacological interventions.

As part of this research collection, Sun et al. showed in a pre-clinical study that the exogenous administration of resolving D1 (RvD1) can prevent sevoflurane-induced cognitive decline by attenuating neuroinflammation in the hippocampus of diabetic rats. The authors showed that the RvD1 proresolution pathway might be impaired in the central nervous system of diabetic rats, which enhances the susceptibility to sevoflurane exposure and leads to exaggerated microglia activity and neuroinflammation that contribute to cognitive decline under type 2 diabetes mellitus conditions.

In the second contribution, Casaril et al. summarized in a mini-review the association between neuroinflammation, mitochondrial dysfunction, and bioenergetic failure in inflammation-associated depression. The authors reviewed clinical and pre-clinical evidence for mitochondrial dysfunction in inflammation-associated depression and how dopaminergic neurons might be particularly vulnerable in this condition. Importantly, the authors addressed sex differences and emphasize the importance of the appropriate inclusion of both sexes in future studies. Of note, the emerging field of immune-neuropsychiatry reinforces that the brain is neither inert nor immune-privileged and can be governed by peripheral immune mechanisms (Dantzer et al., 2021).

Neuroinflammation and oxidative stress can also contribute to the development of cognitive decline. Reis and Castro-Faria-Neto discuss in a mini-review the possible mechanisms that trigger long-term cognitive decline developed because of systemic infectious diseases, such as malaria, sepsis, and SARS-Cov2. Mechanistically, activation of pattern recognition receptors, excitotoxicity, increased generation of ROS, and ferroptosis have been discussed. The authors also provide a detailed description of the crosstalk between neurons and glial cells in physiological and pathological conditions, emphasizing the impact on BBB integrity, and endothelial and synaptic function. However, there is no effective therapy to treat cognitive decline associated with infectious diseases. In the end, the authors emphasize the importance of conducting clinical studies in parallel with improving the experimental models or systems biology approaches to better elucidate the association between severe systemic infectious diseases and cognitive decline.

In an original research article, Zhu et al. describe the existence of distinct phenotypes of inflammation-associated macrophages and microglia in the postmortem dorsolateral prefrontal cortex of control subjects vs. people with schizophrenia through gene expression analysis. Various macrophage markers, but not microglial markers, were increased in patients with schizophrenia, which suggests that macrophages, rather than microglia, might play a bigger role in neuroinflammation in this disease. These promising results open a window for the use of single-nuclei RNA-seq to confirm the inflammatory status of macrophages and microglia in schizophrenia and to anatomically map these changes.

In another original research article, Jang et al. explore the role of the kynurenine pathway and stress in alcohol use disorder and internet gaming disorder in young adults. The authors showed that subjects with addictive disorders showed higher stress levels and lower resilience levels than the healthy control group. Additionally, subjects with alcohol use disorder had higher plasma levels of kynurenine and kynurenine/tryptophan ratio as well as decreases in kynurenic acid and kynurenic acid/kynurenine ratio compared to healthy control subjects. Subjects with internet gaming disorder exhibited plasma levels of kynurenine and kynurenine/tryptophan ratio values intermediate between alcohol use disorder and healthy control subjects. The authors suggest that stress might affect the kynurenine pathway creating a vulnerable neuronal network, which might contribute to the development of addictive disorders.

The exact mechanisms underlying the link between immune-inflammatory activation, oxidative stress, and psychiatric disorders are not fully elucidated and are likely to be complex and disease-specific. Therefore, many different lines of research should be pursued when searching for better treatments or gaining more knowledge about the etiology of psychiatric diseases. Contributing articles to this Research Topic have highlighted some of the biological pathways and potential pharmacological approaches for the treatment and understanding of psychiatric disorders.

## Author contributions

AC conceptualized and wrote the editorial.

## Conflict of interest

The author declares that the research was conducted in the absence of any commercial or financial relationships that could be construed as a potential conflict of interest.

## Publisher's note

All claims expressed in this article are solely those of the authors and do not necessarily represent those of their affiliated organizations, or those of the publisher, the editors and the reviewers. Any product that may be evaluated in this article, or claim that may be made by its manufacturer, is not guaranteed or endorsed by the publisher.
